# Characterization of Tunisian Sun-Dried Merguez: Physicochemical and Compositional Changes During Drying

**DOI:** 10.3390/foods15173160

**Published:** 2026-09-07

**Authors:** Khaoula Belguith, Zeineb Jrad, Amna Chahbani, Mohamed Debara, Talel Bouhemda, Mohamed Hammadi, Halima El Hatmi

**Affiliations:** 1 Physiopathology, Food and Biomolecules Laboratory (LR17ES03), Higher Institute of Biotechnology Sidi Thabet, University of Manouba, Ariana 2020, Tunisia; 2 Livestock and Wildlife Laboratory (LR16IRA04), Institute of Arid Land, University of Gabes, Medenine 4100, Tunisia; jradzeineb@yahoo.fr (Z.J.); amna.chahbani@gmail.com (A.C.); mhammadi70@gmail.com (M.H.); halima.elhatmi@ira.rnrt.tn (H.E.H.); 3 Higher Institute of Applied Biology, University of Gabes, Medenine 4100, Tunisia; 4 Central Laboratory, Institute of Arid Land, University of Gabes, Medenine 4100, Tunisia

**Keywords:** sun-dried merguez, fatty acid, minerals, organic acid, sausage, volatile compounds

## Abstract

The sun-dried merguez sausage (DM) is deeply rooted in the Tunisian culinary culture. It is characterized by a distinctive composition and an energy-saving process. Unlike fermented or mechanically dried meats, sun-dried merguez is produced through a single, natural solar dehydration step lasting 8–12 days. Limited information is available regarding its physicochemical properties, chemical composition, and nutritional profile. To address this gap, changes in physicochemical properties during drying were monitored, and the chemical composition and microbiological flora of fresh merguez (FM) and DM were analyzed. Results showed that sun-drying contributed to a significant decrease in moisture (17.0%), water activity (0.675), and pH (5.56), ensuring microbiological stability and safety. DM was rich in lactic acid (1865 mg.kg^−1^) and other low-molecular-weight organic acids, as well as minerals, particularly iron (13.5 mg per 100 g), with an enhanced fatty acid profile—specifically oleic acid—due to tail fat. The distinctive composition of DM, enriched with 15% spices and herbs, yielded a volatile profile dominated by terpenes (67%), which imparts its characteristic flavor and may contribute to health benefits. The current study underscored the nutritional and nutritive value of DM, offering valuable insights for stakeholders to enhance and protect this cultural heritage.

## 1. Introduction

Merguez is a traditional North African sausage [1,2]. Historically, it was prepared at home during Eid al-Adha (Islamic festival of sacrifice). Several recipes exist, including fresh (FM) and sun-dried merguez (DM), fresh and sun-dried osban [3], and El-Guedid (sun-dried meat) [4]. In the absence of refrigeration, sun-dried products are stored in an earthenware jar at room temperature [5].

Merguez is a spicy sausage made from sheep tail fat and beef or lamb. It is heavily seasoned with dried mint, fennel seeds, and harissa, a chili-based paste with garlic, caraway, and coriander, considered a hallmark of Tunisian cuisine [6]. Merguez sausage is prepared in two forms: FM and DM [5,7].

FM, with a short shelf life, is widely used in traditional dishes such as couscous [8] and ojja [9], or is served grilled in fast-food settings [1,8]. It is sold in markets and butcher shops [9], and is appreciated internationally, including in Europe [1,7]. Several investigations on FM have addressed technological improvements, such as the application of microbial starter cultures [10] and the incorporation of plant extracts [11]. Other studies have focused on biological hazards, including antibiotic resistance [12] and parasitic infections [8].

DM is a long-shelf-life product, traditionally prepared at home during the summer using the same ingredients as FM. It is available in a very limited number of butcher shops or supermarkets or included in traditional dishes at high-end gastronomic restaurants.

The sun-drying of food is an ancient, inexpensive, and sustainable method. It promotes dehydration, reduces weight, concentrates nutrients, enables long-term storage, and imparts a distinctive sensory profile. DM differs from non-fermented dry-cured meats, such as ham and loin, which are made from whole muscle pieces [13]. It also differs from traditional dry-cured fermented sausages such as chorizo [14], traditional Chinese sausages [15], Turkish sucuk [16], etc., classified as fermented dry-cured fermented meat products under the recent ISO 23854 [17], which undergo fermentation followed by ripening or drying. DM is produced through a single sun-drying step lasting 8 to 12 days [18]. It resembles El-Guedid (dried meat) and South African droëwors (a non-fermented dry sausage traditionally dried in sunlight) [19,20,21]. According to the Gagaoua classification [1], DM can be considered a dried, non-fermented meat product. DM, dried Osban and El-Guedid, should be classified as a new category of “non-fermented sun-dried-cured meat products”.

Multiple constraints limit the reproducibility of DM. Safety remains compromised under uncontrolled hygienic conditions that depend on climatic variability. Future research should overcome these limitations. Controlled solar drying technologies such as direct-flow evacuated-tube solar dryers [22] and hybrid solar drying systems [23] could reduce dependence on climate variability and improve food safety. Integrating modern monitoring tools for physicochemical parameters would allow better control of drying kinetics and product stability. To the best of our current knowledge, only one study has described and investigated changes in reformulated dromedary DM, reporting a weight loss of 54.1%, a water activity of 0.673, a pH of 4.97, and a moisture content of 12.3% [18]. No study has examined the composition of the traditional DM made from beef and sheep tail fat. This product is unique due to its high seasoning content and flavor profile enhanced by solar dehydration.

The current study aims to monitor changes in physicochemical parameters during drying and to characterize the chemical composition before and after drying. The objective is to assess the nutritional quality of DM and to elucidate the critical parameters that underpin product stability. The findings may provide valuable insights for scientists, artisans, and the food industry, enabling investment in this promising product by harnessing abundant solar energy, intensified by global warming, while preserving cultural heritage.

## 2. Materials and Methods

### 2.1. Materials and Chemicals

Three batches of FM (3 kg/batch) were made from beef carcasses (*biceps femoris* muscle) and sheep tail fat by three different butchers in Tunis (Tunisia). Spices and herbs were purchased from Epices et Saveurs (Piments Chakroun Chams S.A.R.L., Sfax, Tunisia) and Kamy S.A. (Nabeul, Tunisia), respectively. The sausages were processed using a meat mincer with a 6 mm plate hole size (Model 22RS, Fimar, Villa Veruccino, Italy) and a manual sausage filler (Model LT70R, Fimar, Villa Veruccino, Italy). All chemical reagents used in the analyses were of analytical grade and purchased from Sigma-Aldrich (St. Louis, MO, USA).

### 2.2. Preparation and Sampling

**Figure 1:**
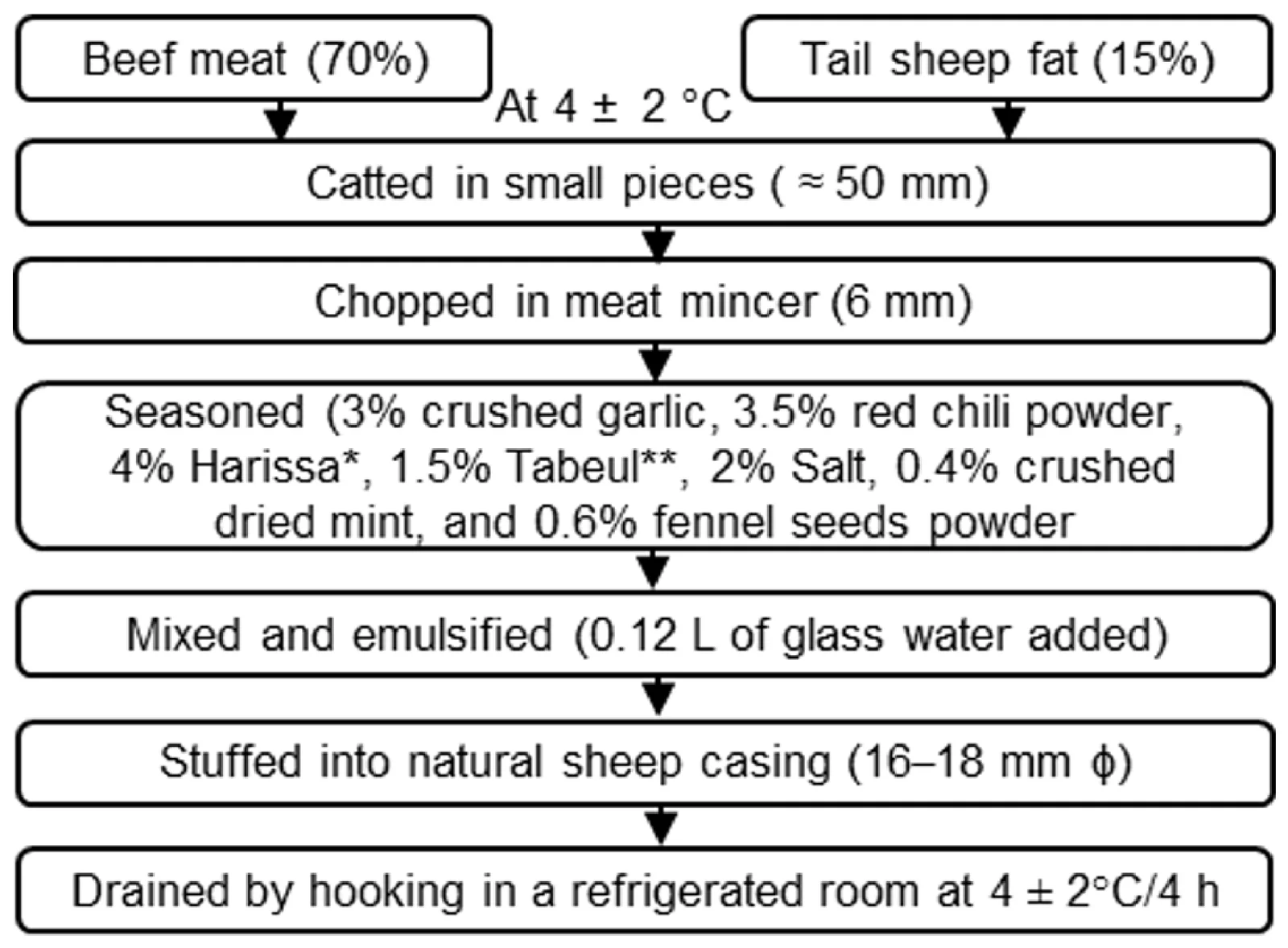
Flowchart of manufacturing artisanal fresh merguez (FM). * Harissa is a type of hot chili pepper paste containing 80% dried chili pepper, 7% olive oil, 4% garlic, 4% coriander, 2% caraway, and 3% salt. ** Tabeul is a Tunisian spice mixture containing approximately 70% coriander, 20% caraway and 10% dry garlic.

The artisanal mixture was formulated and manufactured at the three butcher shops according to the traditional process (Figure 1), and was naturally dried at ambient temperature for 12 days until the weight became constant [18]. In each batch, an initial portion of 2 kg (day 0) was weighed every 48 h throughout the drying period to determine the average rate of relative weight loss. The remaining 1 kg was used for sampling during the process: a first sample (day 0) of approximately 350 g was collected for the different FM analyses. During drying, 20 g samples were taken every 48 h (days 2, 4, 6, 8, 10, 12) for immediate pH and water activity (a_w_) measurements. Finally (day 12), 350 g of DM was collected for subsequent analysis. All analyses were performed within 24 h at 4 °C.

### 2.3. Measurements of Temperature, Relative Humidity, and Average Rate of Relative Weight Loss

The environmental temperature and relative humidity (RH) were recorded daily using an air quality detector (DM306D, Dienmern, Zhongshan, China) equipped with infrared (NDIR) and laser-scattering sensors. The relative weight loss (*r*_wl_) was calculated according to the following equation:

$$r_{\mathrm{wl}}=\frac{\left(m_{i}-m_{i+2}\right)}{48}$$

where *r*_wl_ is the average rate of relative weight loss on day i in g/h, and *m*_i_ and *m*_i+2_ are the weight of merguez for day i and day i + 2 in g.

### 2.4. Measurements of pH and Water Activity

The pH was determined according to ISO 2917 [24] using a WTW™ pH meter (Model 3310, Xylem Analytics, Weilheim, Germany), previously calibrated with standard buffer solutions at pH 4.01, 7.00, and 10.01 (XS Instruments, Via della Meccanica, Carpi MO, Italy). Water activity (a_w_) was measured following ISO 18787 [25] with a NOVASINA^®^aw meter (LabSwift-aw, Novasina AG, Lachen, Switzerland).

### 2.5. Microbiological Analysis

Microbiological analyses were performed before and after drying. A 25 g sample was mixed with 225 mL of buffered peptone water (Chemosolute 8449, Th. Geyer GmbH & Co. KG, Renningen, Germany) and homogenized. A series of decimal dilutions was prepared in tryptone salt broth (BK014HA, Biokar diagnostics, Allonne, France) and inoculated into the following media to enumerate bacterial flora after incubation under specified conditions (temperature/time) according to appropriate standards: plate count agar (70152, Fluka, Steinheim, Switzerland) for mesophilic aerobic bacteria (30 °C/24–48 h) [26]; de Man Rogosa Sharpe agar (CM1153B, Oxoid Ltd., Hampshire, UK) for lactic acid bacteria (LAB) (30 °C/24–48 h) [27]; yeast and molds agar (CM0920B, Oxoid Ltd.) for yeast and mold (25 °C/96–120 h) [28]; violet red bile agar (CM1082B, Oxoid Ltd.) for enterobacteria (37 °C/24 h) [29]; chromogenic tryptone bile X-glucuronide agar (Labkem AGTB-00I-500, Labkem Ltd., Kilbake House, Corballis, IRE) for *Escherichia coli β-glucuronidase+* (44 °C/24 h) [30]; and iron sulfite agar (Condalab Torrejón de Ardoz, Madrid, Spain) for sulfite-reducing anaerobic bacteria (37 °C/48 h) [31]. Enumeration of coagulase-positive Staphylococcus was carried out according to NF V 08 057-1 [32] and the detection of *Listeria monocytogenes* was performed according to ISO 11290-1 [33].

### 2.6. TBARS Assay

Lipid oxidation was assessed using thiobarbituric acid reactive substances (TBARSs) according to Mukombo et al. [20], with some minor modifications. Briefly, 2 g of sample was homogenized in trichloroacetic acid and water, filtered, and added to thiobarbituric acid. After incubation at 80 °C, a pink chromophore was measured spectrophotometrically at 532 nm. TBARS values were calculated using 1,1,3,3-tetramethoxypropan as the standard, and results were expressed as mg malonaldehyde equivalent per Kg dry matter (mg MDA/Kg).

### 2.7. Determination of Proximate Composition

The moisture content was assessed using the oven-drying method at 105 °C for 24 h [34]. The total protein content was determined by the micro-Kjeldahl distillation method using a distillation unit (Digestion Unit PRO-NITRO II, J. Selecta, Barcelona, Spain). The protein content was calculated as N × 6.25. The fat content was determined according to ISO 1443 [35]. Samples were boiled with hydrochloric acid, filtered, dried, and extracted with n-hexane. The carbohydrate fraction and the energy value were calculated, respectively, according to Mossie et al. [36] using the following formulas:

%carbohydrate=100%−(%moisture+%protein+%lipid+%ash)

Energy(Kcal/100g)=(4×Protein)+(9×Fat)+(4×Carbohydrate) 

### 2.8. Mineral Analysis

The dry matter was incinerated at 550 °C for 6 h in an electric muffle furnace, then mixed with 3 mL of a hydrochloric acid solution (1 N) and 3 mL of deionized water. The mixture was subsequently boiled for 5 min and filtered. Mineral contents (Na, K, Mg, Zn, Ca and Fe) were determined by atomic absorption spectrometry (Model AA-6800, Shimadzu Corporation, Kyoto, Japan) in accordance with ISO 6869 [37].

### 2.9. Organic Acid Analysis

Organic acid analysis was conducted according to Dursun et al. [38], with minor adjustments. Sulfuric acid (10 mM) was added to the sample at a ratio of 4:1 (*w*/*v*). The mixture was homogenized and centrifuged at 10,000× *g* for 20 min (4 °C). The supernatant was filtered through a 0.45 μm syringe filter, and 10 μL was injected into the HPLC system (Model UFLC XR, Shimadzu Corporation, Kyoto, Japan). Separation was accomplished using an organic acid column, Hi-Plex H (7.7 × 300 mm, 7.8 μm) (Agilent Technologies, Santa Clara, CA, USA). The mobile phase was sulfuric acid (100 mM). The flow rate was 0.6 mL/min at 50 °C. Organic acids were identified at 210 nm, and their proportions were calculated by comparing retention times and peak areas with those of standard solutions for each acid.

### 2.10. Fatty Acid Identification

Lipids were extracted with hexane/isopropanol [39]. The sample was subjected to methylation using a solution of KOH (2 N) prepared in methanol. The mixture was thoroughly agitated with hexane to extract the fatty acid methyl esters (FAMEs). FAMEs were analyzed by gas chromatography (QP2010, Shimadzu, Tokyo, Japan) coupled with mass spectrometry with a fused silica capillary column, Supelcowax™-10 (30 m length × 0.25 mm i.d., 0.25 µm film thickness; Supelco, Bellefonte, PA, USA). Injection conditions were 250 °C, with a split ratio of 10.0 and a flow rate of 1.20 mL/min. Helium (99.99% purity) was used as the carrier gas. Fatty acids were quantified and identified using the GC–MS solution software, Version D.03.00 (Shimadzu Corporation, Kyoto, Japan) and Wiley 275 mass spectra libraries (Wiley, San Diego, CA, USA).

### 2.11. Volatile Compound Analysis

Volatile compounds were extracted using a static headspace (HS) sampler at 50 °C for 20 min. The headspace volatiles were subsequently injected into the GC in splitless mode with an injection volume of 3 mL. Analyses were performed using a Shimadzu HS-GCMS QP2010 Ultra gas chromatograph coupled to a mass spectrometer (Shimadzu Corporation, Tokyo, Japan) with an RTX-5MS capillary column (30 m × 0.25 mm i.d., 0.25 µm film thickness; Restek Corporation, Bellefonte, PA, USA) [40]. The electron ionization mass spectra were collected via a mass spectrometer with an ion source temperature of 200 °C and a mass range of *m*/*z* 35–500. The volatile compounds of different merguez samples were identified by matching the mass spectra to those in the Wiley 275 mass spectra library (software, D.03.00, California, PA, USA).

### 2.12. Statistical Analysis

All tests were replicated three times and statistically analyzed using XLSTAT (version 2015.5; Addinsoft, Paris, France). Data were checked for normal distribution and homogeneity and then subjected to one-way analysis of variance (ANOVA), followed by Tukey’s honestly significant difference (HSD) test to determine the significance at *p* < 0.05. Data are expressed as the mean value ± standard deviation.

## 3. Results and Discussion

### 3.1. Changes in the Rate of Relative Weight Loss, Water Activity and pH

**Figure 2:**
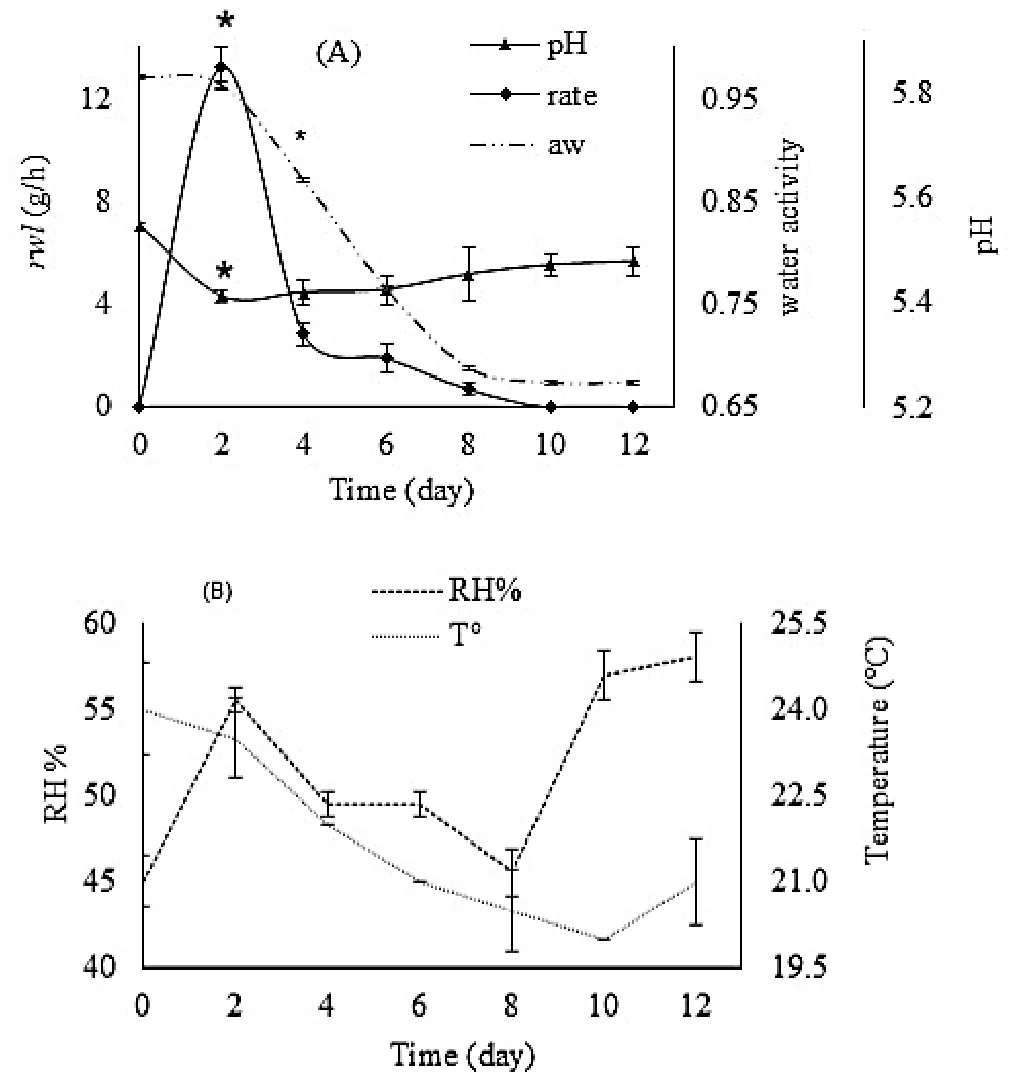
(**A**) The relative humidity (RH) and temperature (T°) of the environment. (**B**) The rate of relative weight loss (*r*_wl_), water activity (a_w_), and pH changes in merguez during drying time. * The mean value exhibits a significant difference (*p* < 0.05) for the same parameter.

After manufacturing, the merguez was dried under ambient air conditions for 12 days. Environmental parameters (RH and temperature), together with *r*_wl_, pH, and a_w_ values of the merguez throughout the drying period, are shown in Figure 2.

During the initial 48 h, a_w_ remained constant, pH decreased significantly, and *r*_wl_ increased markedly. Over this period, RH increased from 45% to 55%, while temperature remained relatively stable (23.0–23.5 °C). By day 4, pH increased slightly (5.50), whereas both a_w_ and *r*_wl_ decreased, with *r*_wl_ reaching zero by day 10 and a_w_ stabilizing at 0.675 (day 8–10). During this interval, temperature dropped to 20 °C, and RH decreased to 45.5% (day 8) before rising to 58% (day 12).

Several factors may explain the *r*_wl_ and pH variation at the beginning of drying (day 0–2). Exposure of fresh sausages to ambient air with relatively low humidity and elevated temperature naturally promotes water release. Moreover, the reduced diameter of FM (16–18 mm) and its high surface-to-volume ratio accelerate dehydration.

In addition, the abundant spontaneous microbiota—including indigenous mesophilic bacteria, LAB, and fungi (Table 1)—combined with nutrient and water availability (Table 2), together with optimal a_w_ and temperature, contribute to marked pH reduction, primarily driven by organic acid accumulation during bacterial proliferation (paragraph 3.5). When the pH decreased to 5.43, which is approximately the isoelectric point of myosin, electrostatic attraction between oppositely charged protein groups reduced the amount of water retained in the protein matrix [41].

Following the initial intense exudation and the progressive reduction in free water availability (a_w_ declining from 0.87 to 0.675), *r*_wl_ decreased, which may have contributed to the development of conditions unfavorable for bacterial growth and further decline.

Based on temperature and RH, South African droëwors is the closest analog to DM, as it is a non-fermented sausage dried at a higher temperature (30–25 °C) and low RH (40–50%). A comparable decrease in moisture was reported after 48 h of drying, despite droëwors’ larger diameter (22 mm) [19,20,21]. Although the processes differ, dry-fermented sausages exhibit comparable drying curves. Their production involves an initial incubation at a relatively high temperature (>20 °C) and a RH of 90–75%, followed by drying at <20 °C and RH < 80%. In these products, the maximum drying rate is typically reached later (day 4). In contrast, DM, processed under lower RH and higher temperature (51 ± 5%, 21 ± 3 °C), undergoes rapid dehydration, resulting in reduced aw, lower moisture content (Table 2), and diminished LAB populations. Conversely, the higher RH and lower temperature conditions in fermented dry sausages slow dehydration, thereby favoring bacterial growth, particularly LAB, and contributing to higher a_w_ and moisture levels.

Regarding drying time, DM dries rapidly (7–12 days), whereas dried-fermented sausages take 12–100 days [13]. Alongside RH and temperature, other factors influence drying rate, a_w_, and pH, such as meat type and fat content and type. Sun-dried camel merguez prepared with camel meat and hump fat, manufactured and dried similarly to DM, exhibited substantial weight loss (>50%), lower a_w_ (0.673), and lower pH (4.97) compared to DM [18]. Mukumbo et al. [20] also reported differences in beef and pork droëwors after 12 days of drying (pH 5.46 vs. 5.63; a_w_ 0.55 vs. 0.60). Fat content is another determinant: Mandela et al. [19] observed that zebra droëwors formulated with 10% sheep fat showed greater weight loss, lower a_w_ (0.878), and lower pH (5.46) compared to those with 20% fat (a_w_ 0.907; pH 5.30). A different perspective emerges when comparing DM (15% fat) with beef sucuk containing 20% fat (12 days of ripening), as DM exhibited lower a_w_ (0.675 vs. 0.891) but higher pH (5.56 vs. 5.08). Ultimately, comparing different sausage types is complex due to multiple interrelated parameters, including seasoning composition, salt concentration, chemical composition, and the distinct autochthonous microbiota ecosystems.

### 3.2. Microbial Changes

During drying, a significant decrease in all counted bacteria was observed (Table 1). The results for *Escherichia coli* (<2.7 log CFU/g), positive coagulase Staphylococcus (<2.7 log CFU/g), and *Listeria monocytogenes* (2 log CFU/25 g) for FM and DM met the regulation limits recognized by European Commission Regulation (EC) 2073 [42] and by the Fédération du Commerce et de la Distribution [43] relative to microbiological criteria applicable to meat and products thereof. Only mesophilic bacteria in FM exceeded the regulatory threshold (>7.7 log CFU/g). However, similarly high initial microbial loads (7.76–9.1 log CFU/g) have been reported by other authors [14,44]. Such a result is necessarily attributable to the processing conditions. Compared to sucuk prepared with sodium nitrite and without a starter [44], the initial counts of mesophilic bacteria, enterobacteria, yeast, and mold in FM were roughly analogous. In contrast, all of these genera were lower in DM, despite DM being a nitrate- and nitrite-free sausage.

DM complied with the regulations, and the sulfate-reducing anaerobic bacteria were reduced to below 1 log CFU/g, supporting the hypothesis regarding the effectiveness of sun-drying. This conclusion remains relative, as several parameters—particularly the climate-dependent nature of the artisanal process—limit its reproducibility.

The small diameter of FM (16–18 mm) promoted dehydration and created unfavorable conditions for microbial growth. Similarly, Nam et al. [45] reported that smaller dry-fermented sausages (18 mm) inoculated with pathogen strains (*E. coli* _O157:H7_, *Salmonella enterica*, and *Listeria monocytogenes*) reached the desired pathogen and a_w_ reduction sooner than larger sausages (30–110 mm). Under one-step dry conditions with low RH (51 ± 5%), the growth of all flora, including yeast and mold, decreased. Comparable results for mesophilic aerobic bacteria (5.3 log CFU/g) and LAB (3.9 log CFU/g) were obtained in Spanish non-fermented dry-cured loin with the same pH (5.5–5.6). Still, yeast and mold were higher (5 log CFU/g) [46], probably due to sunlight action on the surface of DM.

The richness of FM ingredients in spices and garlic plants (15%)—including mint, coriander, caraway, and pepper—promotes pathogen inhibition [47] and provides a natural alternative to nitrite use [48].

As expected, and in contrast to dried-fermented sausage where LAB typically increase, LAB decreased (5.7–3.9 log CFU/g) after drying in DM. Depending on product type, casing size, and processing conditions, the fermentation step in dried-fermented sausages generally lasts between 2 days [49] and 50 days [50], favoring LAB growth, especially when starter cultures are added. During the first two days, extrinsic factors (RH, temperature) and intrinsic factors (a_w_, pH, nutrients) strongly favored bacterial proliferation, particularly LAB. Subsequent drying, with reduced a_w_ and pH, limited growth and explained the observed decline in bacterial counts, including pathogens [51]. Sausages with a pH below 5 and a_w_ below 0.91 are considered shelf-stable products and do not require refrigeration [52]. A water activity of less than 0.84, such as that of DM (0.675), could prevent the growth of food-borne pathogens [53] and the production of ochratoxin [54]. However, the absence of mycotoxin risk remains to be demonstrated. Several mycotoxins, including aflatoxins, ochratoxin A, citrinin, and cyclopiazonic acid, have been detected in dry-fermented sausages [55].

### 3.3. Proximate Composition

The compositions of FM and DM, including moisture, protein, fat, ash, carbohydrate, and energy, are shown in Table 2. A significant decrease in moisture content was observed between FM (60.9%) and DM (17.0%). The drastic dehydration contributed to the increase in all components, especially fats, resulting in a high energy value.

In comparison to the closest sausage, the moisture of beef droëwors was initially comparable; however, after drying, droëwors retained less moisture [19,20], probably because droëwors samples were dried at a higher temperature (25–30 °C) and lower RH (40%) than DM (Figure 2), as explained above. Protein and fat contents are lower than those of droëwors, whereas the ash composition of DM is higher [20]. This effect is most likely explained by the herbs and spices (15% of the contents), which lower the proportion of protein and fat. Compared with other reported compositions of traditional dry-fermented sausages, such as chorizo [56], traditional Chinese sausages [15], and Turkish sucuk [16], DM has lower moisture, protein, and fat contents.

### 3.4. Mineral Composition

**Figure 3:**
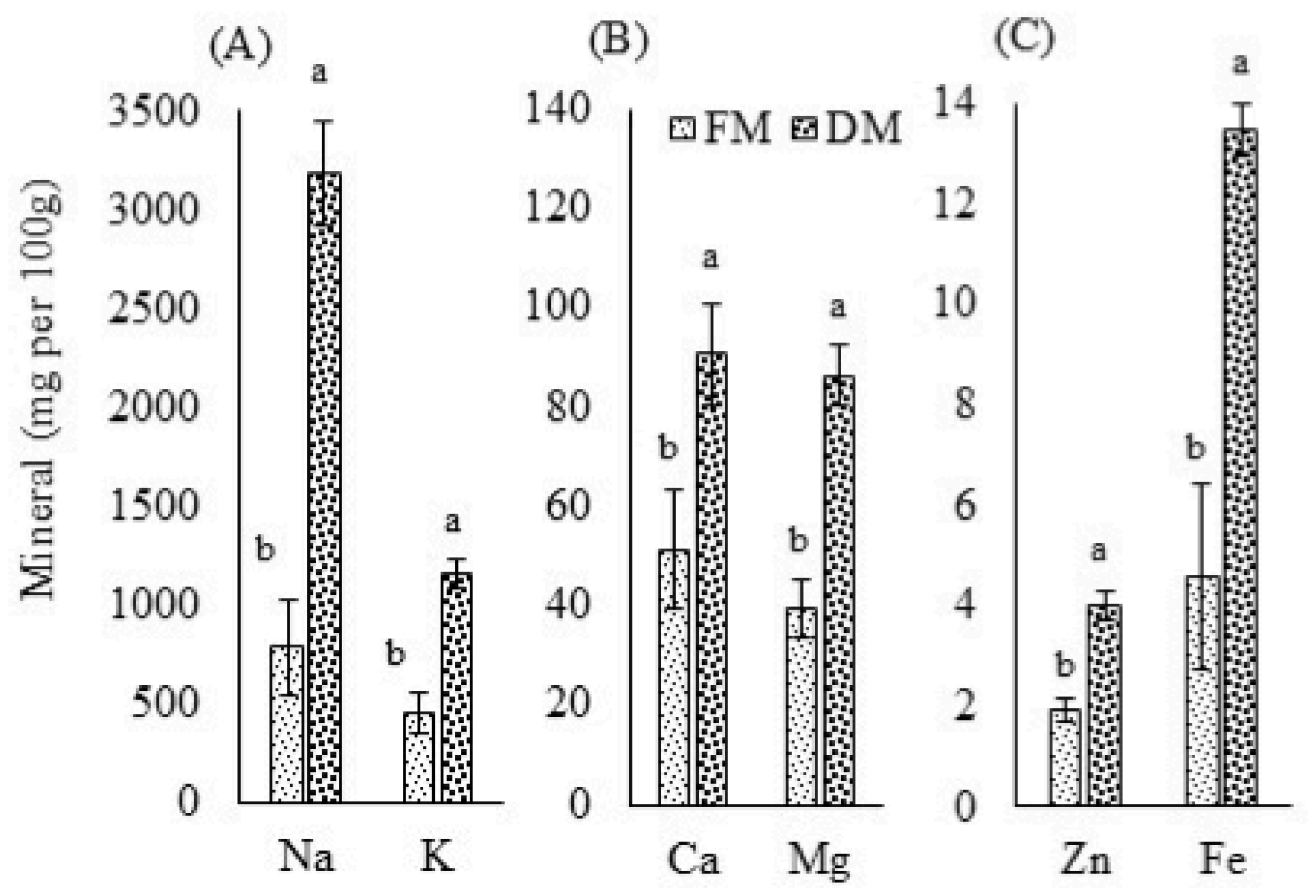
Mineral concentration of sodium, potassium (**A**), calcium, magnesium (**B**), zinc, and iron (**C**) in fresh (FM) and sun-dried merguez (DM). The different lowercase letters a and b indicate a significant difference between FM and DM in each mineral (*p* < 0.05).

The mineral contents of sodium, potassium, calcium, magnesium, zinc, and iron in FM and DM (Figure 3) significantly increased after drying, as expected, due to the concentration of constituents following dehydration. In both forms of merguez, sodium—followed by potassium—was the dominant macroelement, whereas iron was the most important microelement. Compared with chorizo [51], all the studied minerals were higher in DM. Reported mineral concentrations in beef are highly heterogeneous, influenced by factors such as breed, sex, age, diet, water intake, farm management system, environmental conditions, and muscle type [57]. In addition to meat-derived minerals, ingredients such as red chili pepper (Figure 1), used separately and in the harissa (more than 3.5% in the formulation of FM), contributed substantial mineral content: more than 3000 mg of potassium, 123 mg of calcium, 146 mg of magnesium, 2.4 mg of zinc, and 4.9 of iron per 100 g [58]. Coriander and fennel seeds, as *Apiaceae*, had a high levels of potassium and calcium [59].

### 3.5. Changes in Organic Acid Composition

**Figure 4:**
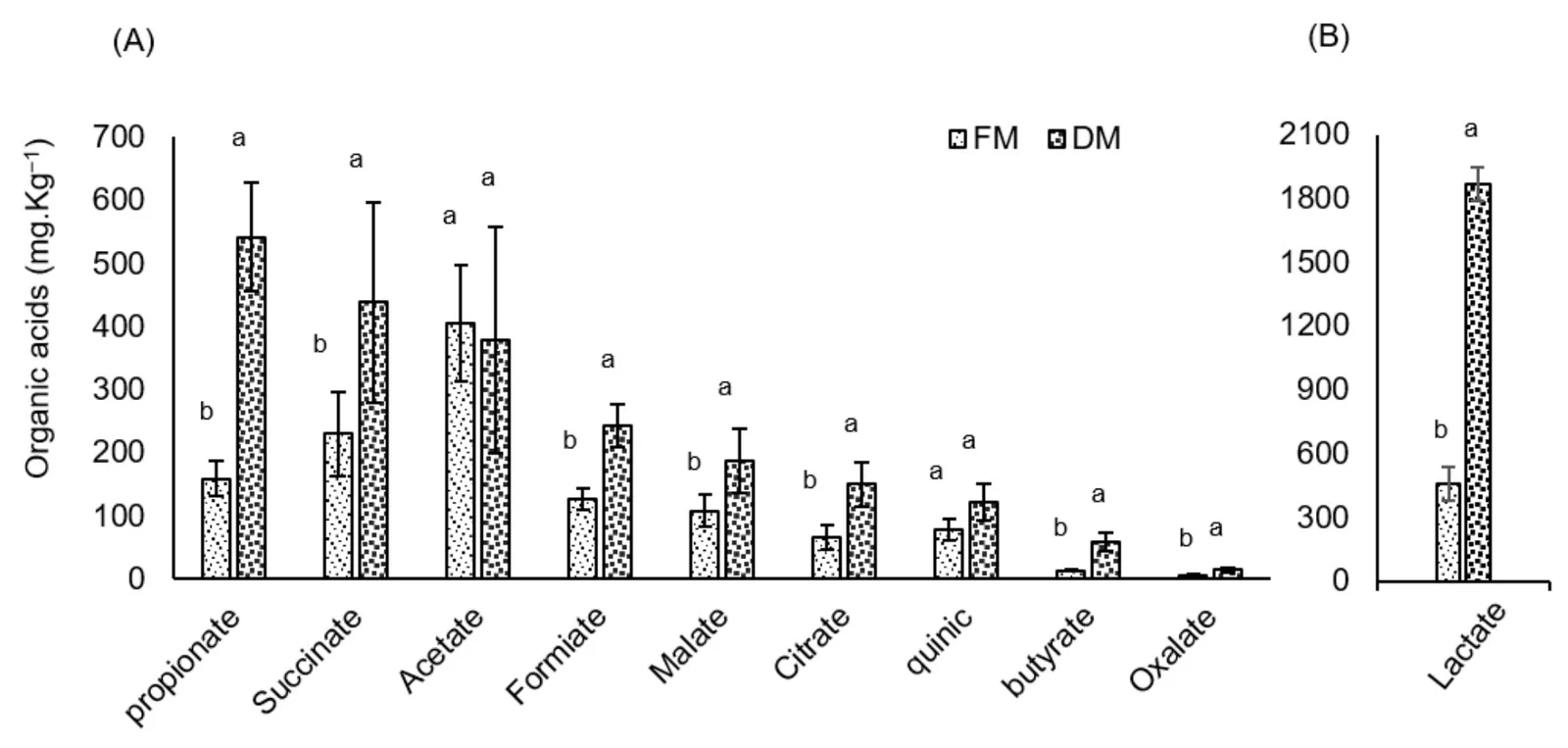
Concentrations of organic acid (**A**) and lactic acid (**B**) in fresh (FM) and sun-dried merguez (DM). The different lowercase letters a, and b indicate a significant difference between FM and DM in each organic acid (*p* < 0.05).

To further explore changes in merguez during drying, the concentrations of ten organic acids (mg.kg^−1^) were determined in FM and DM. Figure 4 shows a significant increase in the concentrations of all analyzed organic acids, except for quinate and acetate, despite the considerable initial concentration of acetate (≈400 mg.kg^−1^).

Lactate was the predominant organic acid in FM, with a level four times higher in DM after drying. These results support the hypothesis that fermentation occurred during the first two days, when conditions were favorable for bacterial growth. During this period, LAB produced organic acids that significantly lowered the pH (Figure 2). In DM, lactate was followed by propionate, succinate, acetate, formate, malate, citrate, quinate, butyrate, and oxalate, in decreasing order.

Li et al. [60] reported the same order of lactic acid and succinic acid in dry-fermented sausage, despite large differences in formulation compared with merguez. They deduced that organic acid synthesis depends on the added starter. Reported concentrations were higher than those in DM, even in non-inoculated sausages, likely due to the use of dextrose, which is rapidly and easily metabolized by autochthonous LAB to synthesize organic acids. The quantification of organic acids in sucuk showed comparable concentrations only in lactic acid. Those of propionic, succinic, malonic, citric, and oxalic acids were higher than in DM, but the propionic acid concentration in sucuk was higher than that of lactic acid [61]. The lactic acid in DM presumably was not converted to propionic acid. Şimşek et al. [62] reported that acetic acid and propanoic acid are common in dry-fermented beef sausages salted with different chloride salt mixtures, with acetic acid lowest in NaCl formulations.

All synthesized organic acids (Figure 4) are widely cited as intermediate metabolites of the tricarboxylic acid pathway of homolactic and heterolactic LAB [60,61]. *Lactobacillus plantarum* has an incomplete tricarboxylic acid cycle, resulting in succinic acid production [62]. Additionally, lactic acid and acetic acid have been identified in the supernatant of cell-free antifungal LAB isolated from dry-fermented sausages [63]. Propionate, whose concentration is tripled in DM, is also widely described as an antibacterial and antifungal agent [64,65]. Beyond pathogen inhibition and spoilage control [66,67], organic acids enhance the sensory and nutritional value of meat products [68] and exhibit antioxidant activity. Machi et al. [69] specifically reported citrate as a scavenger against hydroxyl radicals. Acetate, propionate, and butyrate are short-chain fatty acids (SCFAs) whose bioactivities have been extensively studied. They influence lipid and glucose metabolism, improve insulin sensitivity, and modulate energy balance. Moreover, SCFAs provide multiple health benefits, including anti-inflammatory, immunoregulatory, anti-obesity, antidiabetic, anticancer, cardiovascular protective, hepatoprotective, and neuroprotective effects [70]. Succinate, a bacterial metabolic precursor of propionate, has also been shown to play a role in activating intestinal gluconeogenesis [71]. However, the biological effect depends on the concentration, pH, non-ionized form of the acid, and product matrix.

### 3.6. Fatty Acid Profile

Sheep tail fat (STF) was obtained from fat-tailed Barbarine, a Tunisian breed renowned for its resilience to food scarcity, owing to the considerable fat reserves in its voluminous, rounded tail [72]. STF is commonly used in traditional Tunisian recipes, and its taste is highly appreciated. STF distinguishes traditional sausages from other beef sausages. Therefore, the fatty acid compositions of DM and STF were determined to identify the most prominent fatty acids in DM. Table 3 shows that STF was the main source of unsaturated fatty acids in DM. A similar proportion of unsaturated fatty acids in STF (53.5%) with comparable proportions of oleic and linoleic acids was previously reported by Li et al. [73]. However, dried homemade beef sausage (beef meat and fat) presented a greater proportion of saturated fatty acids (59.10%), particularly palmitic acid [74], compared with DM. This reflects the fact that beef fat contains more saturated fatty acids (63–65%) than STF [75]. The main fatty acid components of DM and STF were C18:1, C16:0 and C18:0, consistent with values reported for beef meat, STF [73], and home beef sausage [74]. Myristoleic acid (C14:1) and eicosenoic acid (C20:1) were detected only in DM, mainly originating from beef meat [73]. Tridecanoic acid (C13:0) was previously detected in mutton sausages [76] and beef sausages [16] but in very low quantities. Using STF instead of beef fat in sausages could improve the fatty acid composition, specifically oleic acid C18:1 (n − 9) and linoleic acid C18:2 (n − 6), which are recognized for their health benefits [77]. In addition, LAB influence the balance of saturated fatty acids (SFAs), monounsaturated fatty acids (MUFAs), and polyunsaturated fatty acids (PUFAs). During fermentation, oleic acid and other MUFAs increase markedly, thereby enhancing the sensory attributes of sausages [78].

### 3.7. Volatile Compounds

**Figure 5:**
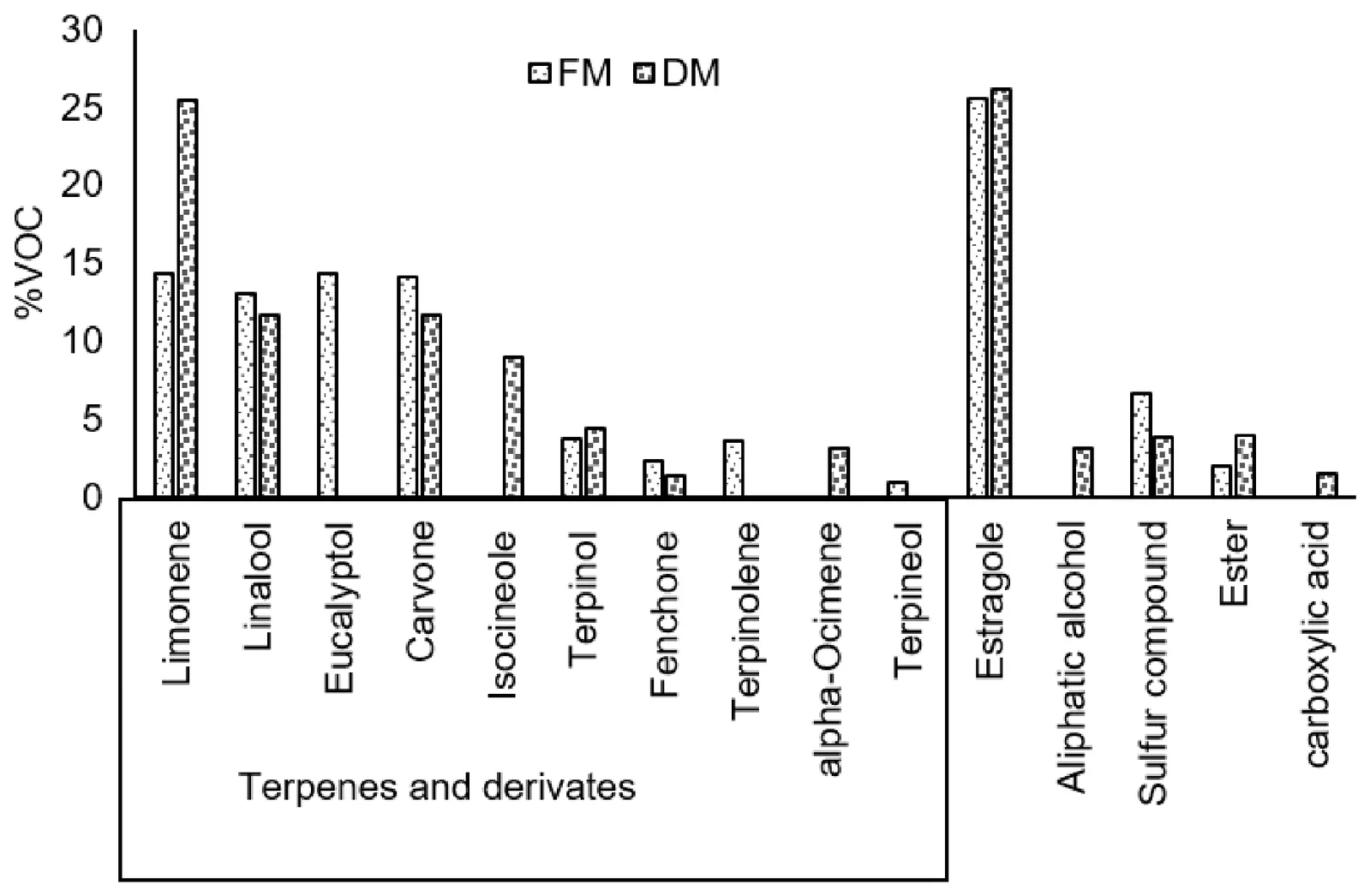
Volatile compounds (VOC %) in fresh merguez (FM) and sun-dried merguez (DM).

A total of 24 volatile compounds were detected and classified into 15 groups according to their functional characteristics. The percentage contribution of each group to the total volatile fraction is presented in Figure 5. The number of compounds identified in FM was higher than in DM. Phenylpropanoids, estragole, and 10 terpenes represented more than 90% of the detected volatile compounds. Zor et al. [79] reported comparable findings in raw meatballs prepared with different vegetable peels. Several studies have indicated that no terpenoids were detected in raw beef [80], dry-fermented sausage [81], or a beef patty [82] without spices. In comparison with other traditional dry-fermented sausage formulations—such as Turkish sucuk (2.5%) [16], Serbian sausage (4.2%) [83], Italian sausage (0.3%) [84], and Iberian chorizo (1.4%) [56]—FM contains 15% spice and herb ingredients, including four different spices and mint (Figure 1).

These ingredients constitute the primary sources of the volatile compounds detected in FM and DM. Limonene, linalool, and ocimene are three terpenes characterized by citrus-like flavors and are predominant in dried chili pepper [85], the most widely used ingredient (in powdered form and as a paste in harissa, as shown in Figure 1) in merguez seasoning. Similarly, caraway and coriander seeds, commonly incorporated into harissa and tabeul (Figure 1), are rich in carvone and linalool. Their essential oils have insecticidal and antimicrobial activities [86]. Mint essential oils are dominated by carvone and limonene [87]. Fennel seeds appear to be the main source of estragole, fenchone, eucalyptol, and terpineol [88]. Beyond imparting their distinctive aromas, terpenes exhibit notable bioactivity, encompassing a broad spectrum of health-promoting effects such as anticancer, antimicrobial, anti-inflammatory, and antiallergic activities [89]. Estragole has strong antimicrobial activity against multidrug-resistant (MDR) bacteria, as well as gastroprotective and anti-asthmatic benefits [90].

The third group of detected volatiles consisted of sulfur compounds, derived primarily from garlic. Li et al. [13] reported that non-fermented dry loins contained the highest levels of sulfur compounds, and alcohols were the predominant volatiles in dry-cured ham compared with fermented dry-cured meat products. This may be analogous to the outcomes observed in DM.

During fermentation, lipids are hydrolyzed to produce free fatty acids, which are subsequently oxidized to generate alcohols, aldehydes, ketones, esters, and other compounds [91]. Despite the presence of esters, aldehydes, carboxylic acids, and aliphatic alcohols synthesized during drying, the total number of detected volatile compounds in DM was less than that reported in dry-fermented sausages [16,92]. The main source of volatile compounds in fermented foods is the metabolic activity of bacterial strains [81,93].

The absence of starter cultures reduced the synthesis of volatile compounds [91]. For instance, under similar conditions (RH ranging from 90 to 80% and temperatures from 22 to 18 °C), Kargozari et al. [16] and Şimşek et al. [92] detected 46 and 60 volatile compounds, respectively, in beef sucuk. The former used *Lactobacillus sakei* and *Staphylococcus carnosus* (7–8 log CFU/kg of sausage batter), while the latter employed higher inoculation levels of *Lactiplantibacillus plantarum* and *Staphylococcus xylosus* (9–10 log CFU/kg). The relatively low number of volatile compounds in DM compared to dry-fermented sausage is likely due to the low RH (Figure 1), which accelerated dehydration and curtailed spontaneous fermentation. This process, carried out by autochthonous microbiota with a low LAB load and no starter culture (Table 1), was further self-limited by decreases in water activity (aw) and pH (Figure 2).

### 3.8. Lipid Oxidation

The TBARS assay (Table 1) showed a low value (<1 mg MDA/Kg) with no significant difference before and after drying, consistent with observations in beef droëwors [20]. TBARS values exceeding 2 mg MDA/Kg are generally associated with noticeable rancidity and reduced sensory quality [94]. Mandela et al. [19] reported that droëwors with 20% fat exhibited the highest TBARS values compared with those containing 10% and 15% fat, while pork droëwors showed higher TBARS levels than their beef counterparts [20]. Furthermore, MUFA appears less vulnerable to oxidative stress compared to PUFA, as indicated by lower TBARS values [95]. Nevertheless, the TBARS assay, which detects lipid peroxidation via MDA, has a threshold of ~1.1 μM, limiting sensitivity in samples with low MDA concentrations. Complementary methods could be used to assess oxidation, such as the peroxide value or conjugated dienes.

In addition to fat content, low lipid oxidation is also linked to merguez ingredients rich in antioxidants and volatile compounds. Terpenes and estragole act as potent radical-scavenging antioxidants, lowering oxidative stress markers such as malondialdehyde (MDA) [89,90]. Akansel et al. [96] observed the lowest mean TBARS value (0.58 mg MDA/Kg) in semi-dry fermented sausage containing 1% fresh garlic. Kim et al. [97] deduced that pH significantly impacted the lipid oxidation of a soybean oil-in-water emulsion supplemented with peppermint extract in the presence of iron. pH reduction through organic acids, in the presence of iron and phenolic antioxidants, may further suppress oxidation in DM.

## 4. Conclusions

The assessment of nutritional quality and physicochemical changes in merguez during drying demonstrated a stable and safe product, with reduced water activity and pH. Its distinctive composition—rich in lactic acid, iron, oleic acid, and terpenes—imparts characteristic flavor and potential health benefits. Sun-drying thus represents a sustainable strategy for producing traditional DM with an extended shelf life. Nevertheless, quality reproducibility is constrained by climatic variability. To improve DM quality, future research should monitor microbiota transformations and select autochthonous lactic acid bacteria to ensure oxidative stability and nutritional quality. Developing controlled sun-drying systems with regulated parameters (temperature, relative humidity, air velocity) will standardize the process and improve reproducibility and safety. With proper hygienic practices, this approach offers strong potential for artisanal and industrial applications, particularly in solar-abundant regions such as Tunisia.

## Figures and Tables

**Table 1:** Fresh (FM) and sun-dried merguez (DM) microbiological parameters in log CFU/g and TBARS in mg MDA/Kg.

Flora (log CFU/g)	FM	DM
Mesophilic aerobic bacteria	8.3 ± 0.2 ^a^	6.2 ± 0.51 ^b^
Lactic acid bacteria	5.7 ± 0. 2 ^a^	3.9 ± 0.1 ^b^
Enterobacteria	4.7 ± 0.2 ^a^	<2 ^b^
yeast and mold	4.7 ± 0.3 ^a^	2.8 ± 0.2 ^b^
*Escherichia coli ß glucuronidase+*	2.4 ± 0.1 ^a^	<1 ^b^
Sulfate-reducing anaerobic bacteria	1.9 ± 0.1 ^a^	<1 ^b^
Positive coagulase Staphylococcus	<2 ^a^	<2 ^a^
*Listeria monocytogenes* per 25 g	n.d	n.d
TBARS (mg MDA/Kg)	0.33 ± 0.01 ^a^	0.31 ± 0.01 ^a^

n.d. = not detected. Means followed by different letters are statistically different (*p* < 0.05).

**Table 2:** Moisture, protein, and fat contents of fresh (FM) and sun-dried merguez (DM).

Components (g/100 g)	FM	DM
Moisture	60.9 ± 0.4 ^a^	17.0 ± 0.3 ^b^
Protein	13.8 ± 0.5 ^b^	22.5 ± 0.3 ^a^
Fat	12.5 ± 0.3 ^b^	24.7 ± 0.6 ^a^
Ash	5.6 ± 0.1 ^b^	9.1 ± 0.1 ^a^
Carbohydrate	7.2 ± 0.7	26.7 ± 0.7
Energy (Kcal/100 g)	196.5 ± 4.4	419.1 ± 6.2

Means followed by different letters are statistically different (*p* < 0.05).

**Table 3:** Fatty acid profile of sun-dried merguez (DM) and sheep tail fat (STF).

%Fatty Acid *	DM	STF
Saturated Fatty Acids		
C8:0	0.026 ± 0.010 ^e^	0.017 ± 0.005 ^c^
C10:0	0.23 ± 0.01 ^e^	0.24 ± 0.03 ^c^
C12:0	0.15 ± 0.09 ^e^	0.16 ± 0.13 ^c^
C13:0	0.04 ± 0.03 ^e^	0.00 ± 0.00 ^c^
C14:0	3.28 ± 0.18 ^d^	2.99 ± 0.09 ^c^
C15:0	2.19 ± 0.53 ^de^	2.00 ± 0.08 ^c^
C16:0	23.22 ± 1.69 ^b^	20.70 ± 3.05 ^b^
C17:0	3.65 ± 1.34 ^d^	3.70 ± 2.61 ^c^
C18:0	18.23 ± 1.92 ^c^	16.64 ± 1.32 ^b^
Total	51.02	46.45
Unsaturated Fatty Acids		
C14:1	2.50 ± 0.06 ^de^	0.0 ± 0.0 ^c^
C16:1	3.72 ± 0.44 ^d^	3.87 ± 1.08 ^c^
C17:1	2.19 ± 0.39 ^de^	1.82 ± 0.37 ^c^
C18:1 (n − 9)	38.00 ± 0.62 ^a^	46.40 ± 10.39 ^a^
C18:2 (n − 6)	2.94 ± 0.08 ^d^	1.43 ± 1.23 ^c^
C20:1	0.21 ± 0.05 ^e^	0.0 ± 0.0 ^c^
Total	49.56	53.52

The different lowercase letters indicate a significant difference (*p* < 0.05). * Most FM results were not detectable or were not repeatable, so only DM results were chosen for consideration.

## Data Availability

The original contributions presented in the study are included in the article; further inquiries can be directed to the corresponding author.
